# Fluorescence-Linked Aptamer Assay for SARS-CoV-2 Spike-Protein: A Step-by-Step Performance Analysis in Clinical Samples

**DOI:** 10.3390/diagnostics12112829

**Published:** 2022-11-17

**Authors:** Pablo Alberto Franco-Urquijo, Mónica Sierra-Martínez, Mariana Jarquín-Martínez, Mateo Alejandro Martínez-Roque, Victor Miguel García-Velásquez, Gustavo Acosta-Altamirano, Nancy Jannet Ruiz-Pérez, Julia Dolores Toscano-Garibay, Luis Marat Alvarez-Salas

**Affiliations:** 1 Laboratorio de Terapia Génica, Departamento de Genética y Biología Molecular, Centro de Investigación y de Estudios Avanzados del IPN, Mexico City 07360, Mexico; 2 Unidad de Investigación en Salud, Hospital Regional de Alta Especialidad de Ixtapaluca, Ixtapaluca 56530, Mexico; 3 Hospital Regional de Alta Especialidad de Ixtapaluca, Ixtapaluca 56530, Mexico; 4 Independent Researcher, Mexico City 07800, Mexico; 5 Unidad de Desarrollo en Soluciones Diagnósticas, Hospital Regional de Alta Especialidad de Ixtapaluca, Ixtapaluca 56530, Mexico

**Keywords:** COVID-19, SARS-CoV-2, spike protein, aptamer, diagnostic test, FLAA

## Abstract

The COVID-19 pandemic has been a main concern over the last two years and has become one of the most important crises in the history of human health. Today, there is still a need for affordable and reliable diagnostic tests for massive disease monitoring. Previously, a set of highly specific DNA-aptamers (C7/C9) binding to the SARS-CoV-2 Spike (S) protein were isolated but its performance in clinical samples remained to be tested. Here, 242 samples were collected through three different methods and subjected to florescence-linked aptamer assays (FLAA) based on C7/C9 aptamers through two readout protocols. Then, a step-by-step statistical approach which included agreement tests, proportion comparisons and binomial and multinomial logistic regressions was used to predict optimal conditions for the novel C7/C9 FLAA test. RTqPCR threshold cycles, symptoms onset and processing time were influential factors on FLAA test results. Naturally occurring mutations on S were also detected and analyzed. Aminoacidic substitutions D614G and T732A appeared relevant for aptamer recognition although further studies are necessary. The methodology presented here is the first step to determine the performance and diagnosis across a range of clinical contexts and it might serve as a base for a complete analysis applicable to other designs of new diagnostic tests.

## 1. Introduction

The timely diagnosis of causative agents in rapidly spreading pandemics is of the utmost importance to monitor and counteract its adverse effects. The current COVID-19 disease sprout caused by the SARS-CoV-2 virus has led to the development of several diagnostic strategies. This disease is presently diagnosed both by clinical symptoms and molecular probing based on nucleic acids, antigen or antibody detection [1]. Some of the now commonly used assays include real-time polymerase chain reactions, immunochromatography, immunofluorescence and/or enzyme-linked immunosorbent assays (ELISA) [1,2], mostly based on the detection of the SARS-CoV-2 virus or in the assessment of the host immune response. Nucleic acid detection by reverse transcription, coupled with a quantitative polymerase chain reaction (RTqPCR) is considered, presently, as the gold standard and is generally designed to amplify one or more SARS-CoV-2 genes; meanwhile, antigen tests are directed to recognize the Spike (S) protein, a well-known highly antigenic structural membrane protein.

Nonetheless, these technologies have some drawbacks, RTqPCR, for example, requires purified RNA, sophisticated equipment and expensive probes, as well as trained personnel, although it is undoubtedly the most sensitive test for the diagnosis of SARS-COV-2 [3,4]. On the other hand, in addition to the complex manufacture, the detection of SARS-CoV-2 antibodies is not appropriate for early diagnosis because they can only be detected between days 7 and 21 post-infection. Additionally, tests based on antibody detection have shown low reproducibility and therefore have not been fully validated [4,5].

Unlike current diagnostic tests, new diagnostic tests for the detection of COVID-19 must be sensitive enough to detect the presence of the virus and thus prevent its spread, as well as being affordable enough and of easy execution to allow multiple repetitions for as many times as necessary. The use of diagnostic technology using aptamers has practical advantages over other technologies [6,7,8].

Aptamers are small single-stranded DNA or RNA sequences that have the ability to bind with high affinity and specificity to molecular targets, generally, proteins that are commonly defined as functional homologs of antibodies. Aptamers are obtained by means of an in vitro iterative selection method called SELEX (Systematic Evolution of Ligands by Exponential enrichment), consisting in a series on Darwinian-like iterative selection cycles which, ultimately, lead to the identification of oligonucleotides that fold into specific secondary structures which allow them to interact with its target protein, whether or not such interaction previously exists in nature [9,10]. 

Several technologies have been applied to perform SELEX, and a relatively recent procedure to obtain aptamers is capillary electrophoresis (Capillary electrophoresis- or CE-SELEX). This method permits the isolation of specific sequences by separating aptamer-target complexes from free oligonucleotides through differences in retention times [11,12,13]. Recently, aptamer recognition of S and N proteins as antigens has been used in the development of a wide range of biosensors due to nucleic acid’s capability to be coupled to nanomaterials employed in optical and electrochemical techniques [8]. Several SARS-CoV-2 aptasensors have been developed, including electrochemical and a variety of optical sensors such as lateral flow assays and aptamer-linked immobilized sorbent assays [14]. Many aptasensors have been tested with a limited number of clinical samples [15,16,17,18]; however, expensive instrumentation or too complicated systems probably limited their potential commercial application [14]. Therefore, aptamer-based biosensor performance analysis under different clinical conditions is needed.

Previously, DNA aptamers have been isolated using ideal filter CE-SELEX (IFCE-SELEX) that rendered two highly specific sequences, C7 and C9, which recognize with high affinity and specificity the Spike protein (S-protein) from SARS-CoV-2 [19]. The detection system developed with the C7/C9 combination has proven to be a reliable design in laboratory-controlled conditions. C7/C9 aptasensor with a calculated 41.87 nM limit of detection (LOD) and a 51 nM sensitivity, also exhibited a low detrimental effect in Spike protein determination from diluted saliva samples with an acceptable recovery range (80–110%) obtained from a Spike-and-Recovery assay. Nonetheless, there was still the challenge of recognition of the S-protein within clinical samples, different conditions and its ability to discriminate among the diversity of SARS-CoV-2 variants

**Scheme 1:**
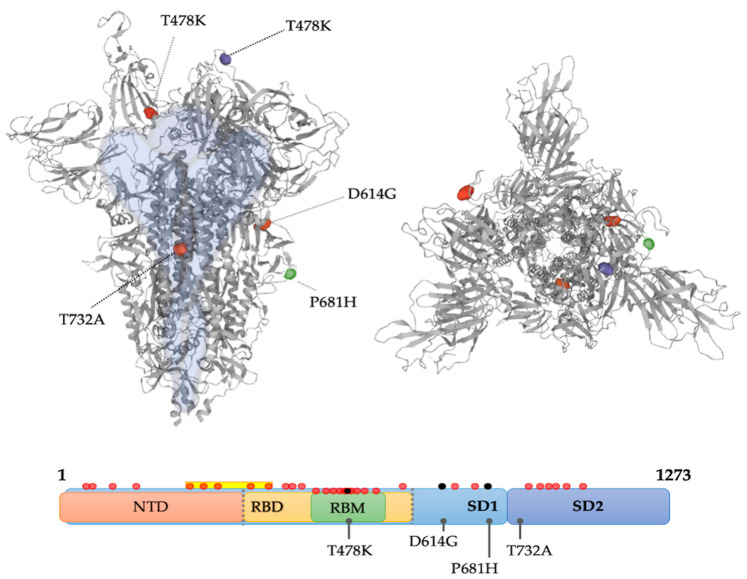
Tridimensional (PDB: 6ZGG) and linear representations of SARS-CoV-2 spike protein. Mutations T478K, T732A, D614G and P681H are presented as red, blue and green volumes. Red dots in linear drawing of S-protein represent sites of reported mutations for Omicron variant. Black circles are detected mutations shared with Omicron variant. Yellow rectangle corresponds to characteristic mutations of delta variant.

Scheme 1 displays the location of some of the S-protein aminoacidic substitutions reported during the observed period. The rendered three-dimensional structure shows that the T478K mutation is found at the upper region of the homotrimer, embedded at the receptor-binding domain (RBD) and at the linear representation it can be seen that is particularly placed at the receptor binding motif (RBM, aa438-506). Mutations D614G and T732A are located equatorially, below the NTD and RBD domains that conform the spicule “head”. In the linear representation, this region is found at the join of subdomains 1 and 2 (SD1 and SD2). P681H is found in an external loop and at the immediate vicinity of the furin cleavage site. All substitutions are exposed and accessible for C7 and C9 recognition.

Here, C7/C9 aptamers were tested on different clinical conditions (mild to moderate disease), sample types (saliva, nasopharyngeal swabs and nasopharyngeal swab preserved in DMEM), preparation protocols (direct or indirect reading), collection and processing times and mutation occurrence, as they came forth in the actual diagnostics laboratory, in order to find the optimal conditions for producing most adequate detection system.

## 2. Materials and Methods

### 2.1. Oligonucleotides 

The 5′-FAM-C9 aptamer was purchased from T4 oligo^®^ (ADN Artificial S. de R.L. de C.V., Irapuato, México). 5′-amino-C6-modified C7 aptamer was purchased from Integrated DNA Technologies Inc. (San Diego, CA, USA). 

### 2.2. Sample Collection and Pretreatment

Samples were acquired by the Research Direction staff of Hospital Regional de Alta Especialidad de Ixtapaluca from consenting healthy and symptomatic volunteer subjects who attended the molecular diagnosis laboratory and/or intermediate care unit. Patients arrived under a variety of conditions including time of symptoms onset, viral variant and loadings. Samples were processed either immediately or saved for a weekly reading program. The collection was performed by standardized day-to-day methods. Briefly, for saliva (Sal) sampling, subjects were asked to allow liquid to accumulate on the mouth floor and then to spit it into a 15 mL polypropylene test tube until enough volume was collected (3–5 mL). During the collection process, sample tubes were kept on ice. To eliminate high viscosity residues, samples were cleared for 3 min at 1500× *g*. Pellet was discarded, and the supernatant was diluted in TNa7 buffer (50 mM Tris-HCl pH 7.0, 110 mM NaCl, 1 mM MgCl_2_) as described in the following section. Nasopharyngeal samples were collected by inserting sterile minitip-swabs through the nostrils of patients placed with their heads tilted at 70 degrees. The swab was slipped parallel to the palate until resistance was found and then gently rolled and rubbed to absorb secretions. Samples were placed in 5 mL of either TNa7 (Sw) or DMEM (Sw-D). After swab removal, the remaining volume was cleared by centrifugation for 3 min at 1500× *g* and supernatants were either immediately processed or frozen (−80 °C) until further use. 

### 2.3. Fluorophore-Linked Aptamer Assay (FLAA)

The FLAA test for SARS-CoV-2 S-protein detection was set in 96-well microplates as previously described [14]. Briefly, 100 pmole of 5′-amino-C6-modified C7 aptamer was immobilized on clear or black opaque Pierce™ maleic anhydride activated plates (Thermo Fisher Scientific, Waltham, MA, USA) as a capture agent. Plates were blocked using the Super-Block™ reagent (Thermo Fisher Scientific) following the manufacturer’s protocol. Supernatant from Sal, Sw or Sw-D samples was added in TNa7 for the final 10% and 20% concentrations; a volume of 100 μL of each dilution was then poured into C7-coated plates and incubated in an orbital shaker for 1 h at room temperature. Wells were washed five times with TNa7 and then 50 pmole of the 5′-FAM-C9 aptamer were added as a detection agent. After a second incubation for 1 h at RT, plates were washed five times with TNa7, and fluorescence was either acquired immediately (direct protocol) from black opaque plates or incubated in clear plates with 150 μL of 7 M urea for 30 min at RT for C9 aptamer retrieval and posterior transference into black plates (Corning Inc., Corning, NY, USA) for further reading (indirect protocol). Finally, fluorescence in the 96-well black plates was measured at 491ex/516em nm using a BioTek^®^ Synergy™ H4 Hybrid Multi-mode microplate reader (Thermo Fisher Scientific).

### 2.4. Mutation Analysis

The presence of SARS-CoV-2 in samples was performed with COVID-19 Real-Time Multiplex RT-PCR Kit (Labsystems Diagnostics Oy, Vantaa, Finland) following the manufacturer’s instructions and using a CFX96 Touch Real-Time PCR Detection System (BioRad Laboratories, Hercules, CA, USA). Mutations T478K(TK), D614G(DG), T32A(TA) and P681H(PH) were amplified from confirmed positive samples with the MUT6 SARS-CoV-2^®^ kit (GENES2LIFE S.A.P.I. de C.V., Irapuato, México) using the same thermocycler. This kit provides a modular disposition based on probe mixes tagged with HEX, FAM, Quasar670 or Cal Fluor Red 610 that specifically distinct the four mutations at different alignment temperatures (TK + DG = 64 °C, PH = 69 °C and TA = 67 °C). The presence of the Omicron variant (B.1.1.529) was determined from positive samples using the MASTER MUT confirmatory O kit^®^ (GENES2LIFE)

### 2.5. Normalization

Two samples from confirmed uninfected patients were added as controls for each plate. Fluorescence normalization was achieved by subtracting the mean signal of controls from sample fluorescence (Sal, Sw or Sw-D).

Normalization of threshold cycles was achieved by correcting the Ct of sample fragments with respect to the ratio of sample internal control (IC) and mean of all IC Ct values, as previously described by El-Malah et al. [20] through Equation (1):

nCt = sCt × (ICCt/mean sICCt)(1)

where nCt is the normalized Ct for each gene, sCt is the sample threshold cycle of genes Orf1ab, N or E and sICCt is the threshold of internal control of each sample.

### 2.6. Diagnostic Accuracy Analysis

The parameters for diagnostic accuracy were determined from a tetrachoric table using the following equations.

Sensitivity = [TP/(TP + FN)] × 100(2)

Specificity = [TN/(TN + FP)] × 100(3)

Accuracy = [TN + TP/(TN + TP + FN + FP)] × 100(4)

Negative predictive value (NPV) = [TP/(TP + FP)] × 100(5)

Positive predictive value (PPV) = [TN/(FN + TN)] × 100(6)

### 2.7. Statistical Analysis

Statistical analysis was divided into three main steps: inner consistency, data distribution and diagnostic accuracy estimations. All data were treated using Jamovi software, version 2.2.5 (https://www.jamovi.org/) [21]. Bland–Altman analysis was used to estimate agreement between measured variables. Scatter plots were used to visualize data distribution and to test preliminary correlations. The Binomial proportion test and Binomial and Multinomial logistic regressions were performed to estimate probabilities of positive results and to construct ROC curves and estimated marginal means plots, respectively. Fagan’s nomogram was employed to test the ability of C7/C9 aptamer system as an auxiliary diagnostic tool for gold standard results.

## 3. Results

**Figure 1:**
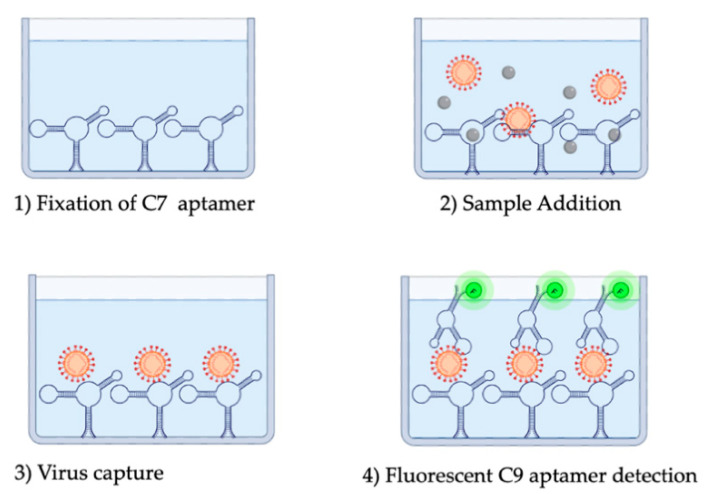
Design of the FLAA assay for SARS-CoV-2 S-protein detection.

Fluorescence-linked C7/C9 aptamer assay (FLAA) was set in a sandwich-type detection system. Briefly, C7 was immobilized on the surface of maleic anhydride-activated 96-well plates as a capture agent. Samples were added to the C7-containing plates, incubated to allow SARS-CoV-2 capture and washed with the TNa7 buffer prior to the addition of fluorescein-labeled C9 as detection agent (FAM-C9) (Figure 1). After a second incubation, multiwell plates were washed and either read directly (black plates) or incubated with 7 M urea before fluorescence transference to black opaque plates.

Under such design, a total of 242 collected samples were acquired as they arrived at the diagnostics laboratory facilities, from mild to moderate symptomatic COVID-19 ambulatory patients. Most samples were obtained before SARS-CoV-2 confirmation by RTqPCR in order to keep a blind procedure. The mean age of participants was 40.5 years old and sex distribution for both genders was similar (*p* = 0.222) (Table 1). Samples were collected in a mean of 5.5 days after the onset of symptoms and processed within 3 days from collection.

### 3.1. First Step: Inner Consistency

To determine the quality of test execution, the mean coefficient of variation was calculated for FLAA (0.026) and for RTqPCR (0.048). The agreement between duplicated fluorescence readings was analyzed, as well as whether the same amount of RNA was used during RTqPCR detection for different SARS-CoV-2 genes. Identification of data between confidence interval margins was performed by means of Bland–Altman plots. Supplementary Figure S1 shows that the FLAA assays were reproducible since the normalized fluorescence values (FLAA1 and FLAA2) remained between the upper and lower limits (Figure S1a). The nCt values for the different viral genes also remained within the confidence zone; Cts from Orf1ab were concordant with those of N (Figure S1b) and E (Figure S1c) genes. Additionally, the nCtOrf1ab remained completely within the confidence interval when compared with the internal control (CtIC, Figure S1d). With these observations, nCtOrf1ab was used as a quantitative parameter of RTqPCR for comparison against fluorescence values in the following analyses.

### 3.2. Second Step: Data Distribution for Preliminary Visualization

**Figure 2:**
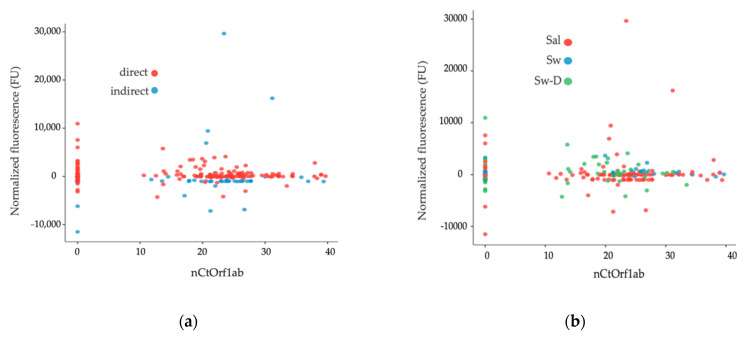
Distribution of fluorescence readings in tested samples. (**a**) Scatter-plot colored for direct and indirect protocols; (**b**) Scatter-plot colored by sample type.

The distribution of fluorescence values with respect to nCtOrf1ab was analyzed in a scatter plot, distinctively indicating those acquired in each experimental protocol and sample type. Figure 2 shows the resulting plot as a duplicate but marked with colors according to each variable. It can be seen that most of the samples that were read with the direct technique have positive fluorescence values and are apparently comprised within a range of nCts between 15 and 30 (Figure 2a). On the other hand, samples processed with the indirect protocol showed low acquisition signals at FLAA, despite belonging to the same Ct range. Regarding the sample types, fluorescence values from swabs preserved in DMEM (Sw-D) corresponded to a more heterogeneous series of nCtOrf1ab (green dots) as compared to saliva (red dots) and swabs in TNa7 (Sw, blue dots) (Figure 2b). It can also be seen that the fluorescence of Sw samples corresponded to amplifications after cycle 20.

Samples from indirect readings had lower fluorescence levels than those processed in a direct manner (Figure S2a). This phenomenon was also observed in the amount of RNA detected by RTqPCR, this is to say, indirect samples presented lower nCtOrf1ab (Figure S2c). Sw-D samples had the highest fluorescence readings but also corresponded to the lowest nCtOrf1ab (Figure S2b). These observations might be explained due to DMEM autofluorescence or because there is more virus present and probably more spike protein. It was also noted that the TNa7 swabs fluorescence was close to the average but had lower amounts of viral RNA than Sw-D samples; this means that, although Sw samples had slightly less virus, the acquisition of fluorescence was acceptable (Figure S2b,d). Finally, the saliva has low fluorescence and also corresponds to low nCtOrf1ab. The difference could have been based on the concentration of samples from 10% in Sal to 20% in Sw/Sw-D in addition to the transference step of C9-fluorescent aptamer between clear and black plates in the indirect reading protocol.

**Figure 3:**
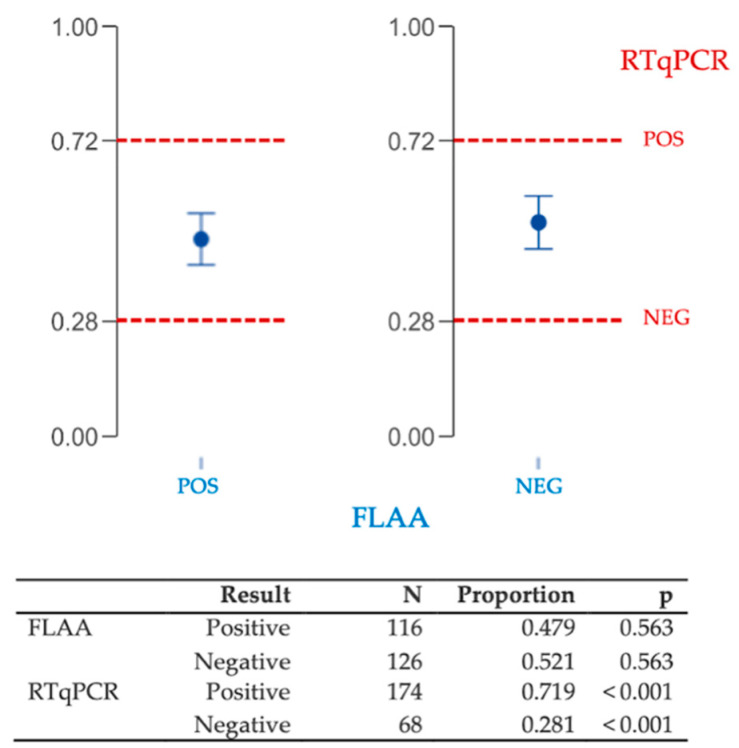
Binomial test for RTqPCR and FLAA positive and negative proportions. No discriminative probability was 0.5, *p*< 0.05 were considered significant.

Subsequently, to determine if the average acquired fluorescence was related to the classification of cases as positive or negative, a binomial test was initially performed in which it was observed that only the RTqPCR results were significantly different from 50% (Figure 3). The proportion graph shows the actual ratio of positive and negative cases classified by RTqPCR (red values), and it can be seen that it significantly differs from the proportion on the FLAA test. In an ideal condition, these numbers in the new test should be equal to the values of the gold standard, this preliminary observation indicated that in a simplified analysis like this, the FLAA could probably show a limited discriminatory ability without considering other variables.

**Figure 4:**
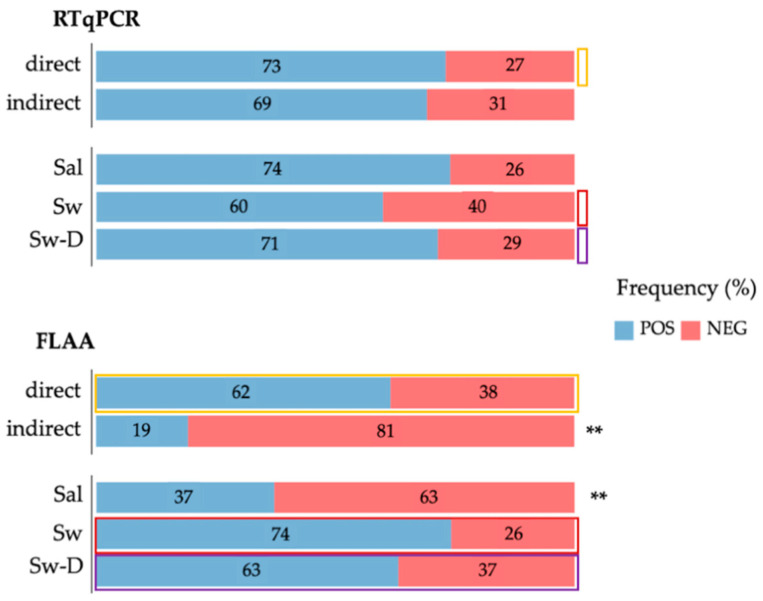
Frequency plots for positive and negative samples percentage divided by protocol and sample types. Yellow, red and purple rectangles represent proportions that should be the same as RTqPCR. ** represent most varying frequency ratios.

**Figure 5:**
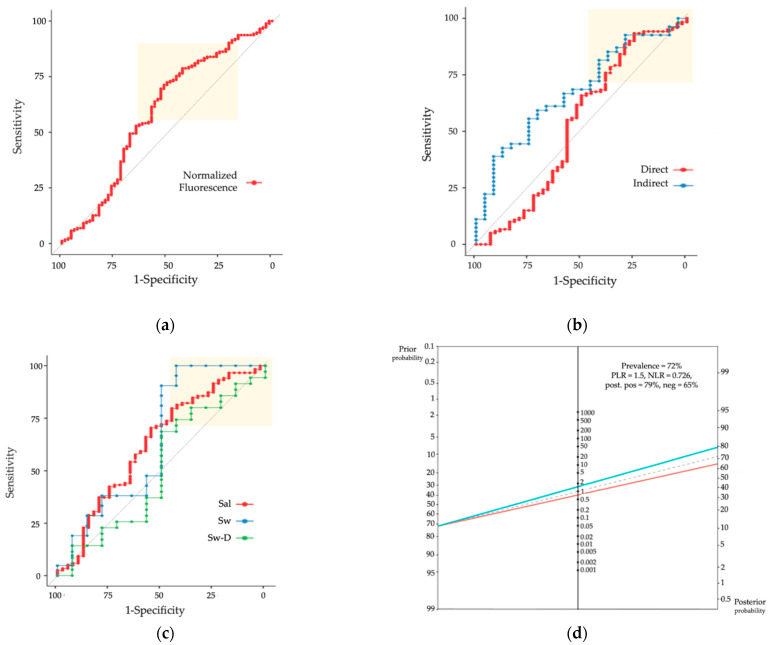
ROC curves for (**a**) overall samples; (**b**) protocol; and (**c**) sample type. (**d**) Fagan nomogram for post-test probability of positive (red line) and negative outcomes (blue line). Yellow squares delimit cutoff zones with high sensibility and specificity.

Using a similar analysis, the proportions of positive and negative cases were broken down and expressed as percentages in frequency plots (Figure 4) for each experimental condition used. The proportions of positive and negative cases were classified with RTqPCR, which correspond to the ideal result, are shown in the upper panel and the FLAA classification can be seen in the lower panel. Again, bars in the FLAA test should be as similar as possible to those in the RTqPCR. It can be seen that the proportion of FLAA in the direct protocol is more similar to that of RTqPCR (yellow rectangle), while the swab samples (red rectangle) are better than those of saliva with a slight advantage of the D-swab (purple rectangle) (Figure 5).

With this simplified descriptive analysis, it was possible to think a priori that the best conditions to read the fluorescence from the C7/C9 aptamer system were with swab samples transported in TNa7 buffer or DMEM and diluted to a 20% *v*/*v* dilution for incubation and direct reading on black opaque plates.

### 3.3. Third Step: Diagnostic Accuracy Analysis by Binomial Regression

In the previous descriptive analysis, the behavior of the FLAA test in the context of different clinical conditions was preliminarily described. However, the most accepted and frequent way of comparing two diagnostic tests is by calculating two main parameters: *sensitivity* (defined as the ability of a new test to designate diseased cases as positive) and *specificity* (understood as the ability of a new test to designate non-diseased cases as negative) [22]. Usually, these parameters are determined considering a single discrimination limit (cut-off point), and by a contrast to the number of positive and negative cases classified through a gold standard. For this case, a tetrachoric frequency table was constructed (Table 2) using zero of the normalized fluorescence signal (0 Fluorescence Units or FU) as the cutoff point.

From this table, a number of true positives (92), true negatives (43), false positives (25) and false negatives (82) were obtained and then the contrast parameters were estimated as described in Material and Methods (Equations (2)–(6)). Globally, the sensitivity of the FLAA test was close to 53% while it had an approximate specificity of 63%, that is, it is a better system for the correct detection of negative cases rather than positive ones, and it performs with an accuracy of 56%. In contrast, the positive and negative predictive values (79% and 34%, respectively) and the positive and negative likelihood ratios (1.43 and 0.745), showed that the magnitude of improvement in the diagnosis of positive cases by RTqPCR could be increased 1.4 times when FLAA test also results in positive cases (Table 2).

As stated before, estimations of sensitivity and specificity were obtained considering readings above 0 FU as positive samples; nonetheless, these values could be recalculated by changing the cutoff and then building a decision model for the classification of positive and negative cases. One way to summarize all the possible existing estimations within a dataset is through the construction of ROC curves (Receiving Operator Characteristics curves). In such graphical representations, different cutoff points give rise to several combinations of sensitivity and specificity, and through the use of the Youden index, the best predicted experimental conditions can be found in order to designate results as close as possible to the actual disease conditions.

Hence, a ROC curve was elaborated for overall samples and is shown in Figure 5a. The zone of best cut-off points for sample analysis was highlighted (yellow rectangles) which corresponds to the farthest zone from the diagonal line (null diagnostic value).

In the same way, Figure 5b,c show the resulting curves of sensitivity and specificity grouped by protocol and sample types. The comparison between protocols indicates that the area under the curve (AUC) was wider for the indirect readings (Figure 5b); however, sensitivity values were around 50%. In practice, it is preferred to have a high number of true positives, so it was determined that the best theoretical pair was obtained at near −404 FU within direct readings. Regarding sample type, AUCs showed that nasopharyngeal swabs collected in TNa7 had the highest sensitivity values (100%) with similar fluorescence readings (−398 FU). The best sensitivity and specificity pairs for each experimental condition are summarized in Table 3.

The degree of validity of the FLAA test as a diagnostic aid for the RTqPCR results acceptable was estimated by the construction of a Fagan nomogram, using prevalence (72%), PPV (0.78) and NPV (0.41) for the overall sample (Figure 5d). In the diagram, the post-test probability of an accurate positive diagnosis was projected to be 75% and as high as 65% for a negative diagnosis (intercepts of red and blue lines with the right axis, respectively) making it 1.5-times more probable for a patient to have COVID-19 when combined with a positive RTqPCR result. This augmentation of the post-test probabilities was consistent with what would be expected in highly prevalent diseases [23].

### 3.4. Fourth Step: Analyzing Influential Factors by Multinomial Regression

The relatively low specificity values led us to think that there were other factors influencing the ability of C7/C9 aptamers to recognize S-protein in addition to the specimen and readout types. One of these possible variables is the timing at which samples were acquired and processed, meaning the time elapsed from symptoms onset and the number of days deferred until fluorescence readings. Both periods might have had an influence on the FLAA results, on one hand, because the amount of virus naturally varies among patients and, on the other, due to possible instability of detectable S-protein after the storage periods.

**Figure 6:**
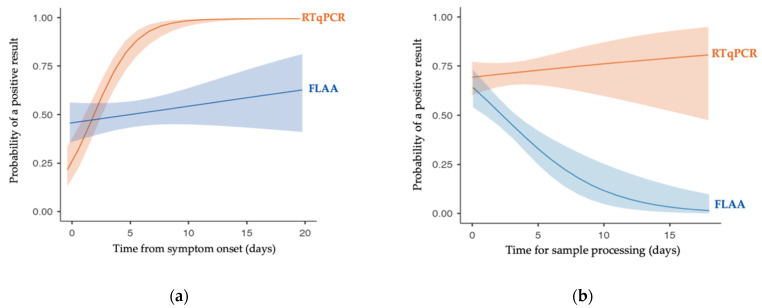
Estimated marginal means plots for (**a**) time from symptoms onset and (**b**) time for sample processing.

To evaluate such an effect, binomial logistic regressions were performed for both FLAA and RTqPCR, and graphs of estimated marginal means were constructed. The probability of obtaining positive results was contrasted to time periods and it can be seen that, in this particular set of patients, the probability of positive RTqPCR results increased gradually and reached the maximum values apparently around 6–7 days after symptoms onset (Figure 6a). This behavior also occurs for the FLAA test but in a moderate way (Figure 6a). Meanwhile, the probability of RTqPCR remained practically constant at all times and noticeably decreased for FLAA even from the first storing day (Figure 6b). These results confirm that extracted nucleic acids are better preserved than proteins when frozen and that, in the case of the C7/C9 aptamer detection system, immediate sample processing might be beneficial.

**Figure 7:**
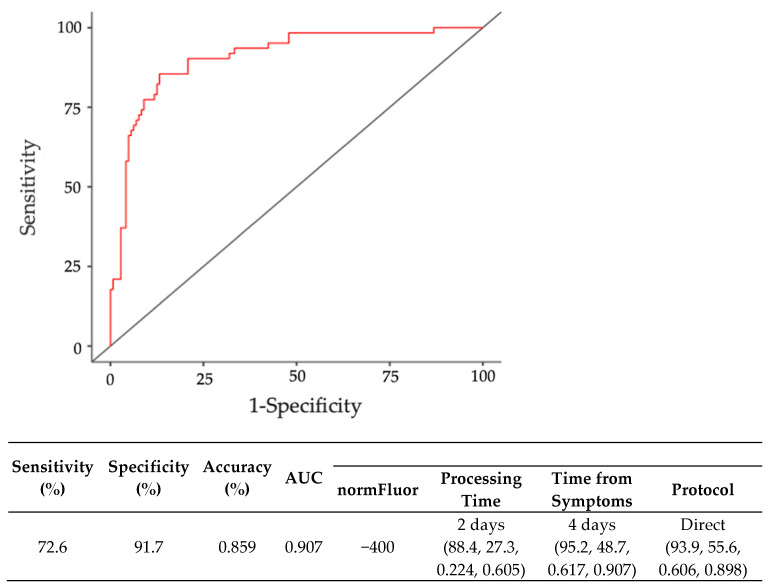
Recalculated ROC curve using all cut-off points found for experimental and time variables. The numbers in parentheses correspond to (Sensitivity, Specificity, Youden index, AUC). Red line: ROC curve, Black line: non-discrimination line.

**Figure 8:**
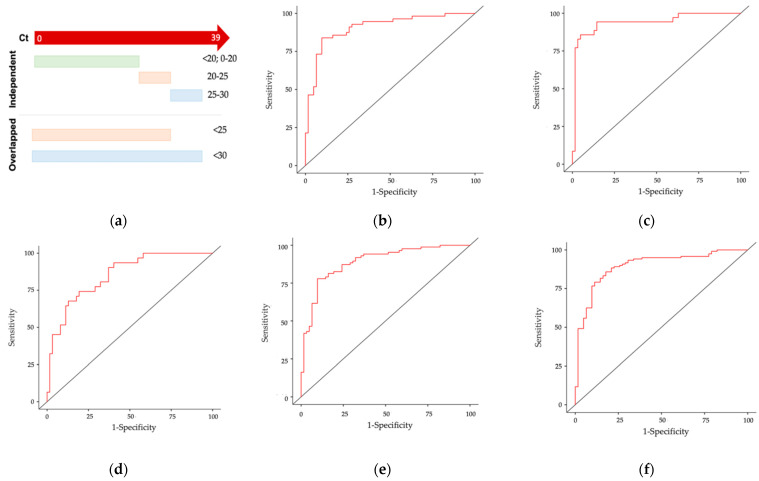
Recalculated ROC curves categorized by nCtOrf1ab ranges. (**a**) Scheme of Ct ranges used for curves adjustment, Red arrow: Ct scale, for independent, green bar: <20;0–20, pink bar: 20–25 and blue bar: 25–30; for overlapped, pink bar: <25, blue bar: <30. (**b**) ROC curve for Ct = 20–25, (**c**) ROC curve for Ct = 25–30 (), (**d**) ROC curve for Ct < 20, (**e**) ROC curve for Ct < 25 and (**f**) ROC curve for Ct < 30. Red lines: ROC curves, Black lines: non-discrimination lines.

Based on previous observations, optimal cutoff points for aptamer performance were obtained from a recalculated ROC curve (Figure 7) using normalized fluorescence, symptoms onset and processing times as covariates; and protocol/sample types as factors. Figure 8 shows that AUC increased up to 91% when using the following conditions: samples should be obtained within the first 4 days of symptoms onset using nasopharyngeal swabs, preserved in TNa7 and processed no more than two days from collection. Additionally, the discrimination cutoff for accurate diagnosis should be considered at −400 FU, after a direct reading on opaque black plates.

Despite these adjustments, the FLAA test would have a sensitivity lower than its specificity (72.6% vs. 91.7%), which possibly indicated that other variables could have an influence on results. Previously, it has been reported that Ct values from RTqPCR determinations have a direct correlation with the capability of new tests to accurately predict positive and negative outcomes [24]. It is generally accepted that lower Ct values are a semiquantitative indication of a high viral load, this is to say, the more nucleic acid detected the higher the number of virions present in a sample and, hence, of detectable protein for antigenic tests (such as FLAA). Then, if a sample has a low Ct value the probability of being detected as a positive case is also higher and the sensitivity and specificity may vary. Hence, sample data were regrouped according to amplification thresholds. Five ranges were considered: samples amplifying under 20, 25 and 30 cycles (overlapping ranges) and from 0–20, 20–25 or 25–30 cycles (independent ranges) (Figure 8a). Then, ROC curves considering the above-mentioned conditions were recalculated for each range. Figure 8b–f shows the resulting curves and it is noticeable that, in fact, Ct differences produce perceivable changes in the form of each curve.

Recalculated diagnosis parameters for aptamer performance at each Ct range are summarized in Table 4. The best pairs of sensitivity and specificity estimates were found in the ranges from 20–25 and 25–30, which were close to those for RTqPCR, suggesting that there could be another factor weighting for the high rate of false negatives.

The samples of this cohort were collected between June 2021 and February 2022; at that time at least two contagion waves had occurred and multiple SARS-CoV-2 variants were detected in Mexico and worldwide.

To estimate whether some of such naturally occurring variants of S-protein could be related to the alteration of the discriminatory ability of our FLAA test, we searched four common mutations using RTqPCR, most of which were frequent in viral variants on this period and were known to cause amino acid changes within spike domains. Briefly, S-protein mutations detected in the samples were: T478K (TK), D614G (DG), P681H (PH) and T732A (TA) in combination, thus forming groups DG, DGTK, DGPH, DGTKTA and DGTKPHTA. Additionally, the deletion of the Orf1ab gene (D2, Δ15:243–257) and the presence of the Omicron variant (B.1.1.529) were also considered. B.1.1.529 contained besides DG, TK and PH, the mutations ΔG142, ΔV143, ΔY144, Y145D, ΔN211, L212I and R214_D215insEPE.

**Figure 9:**
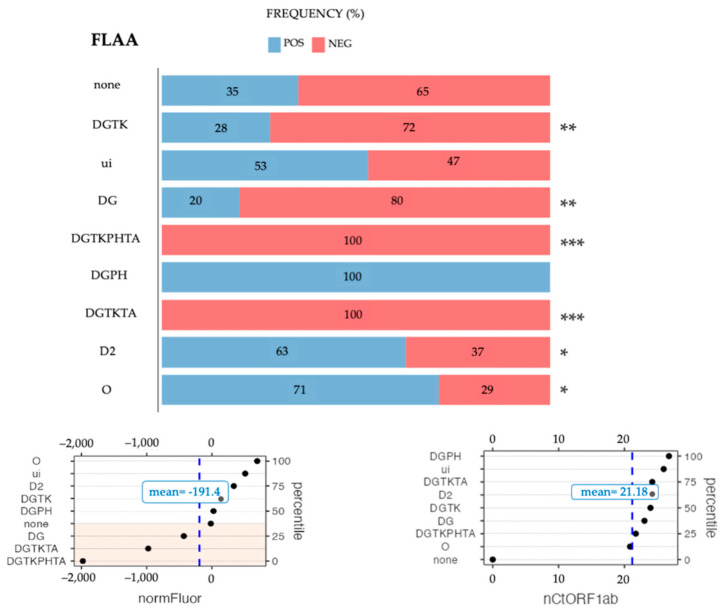
Frequency plot and dot-plots for means of normalized fluorescence and CtOrf1ab. * = moderate proportion variability; ** = high proportion variability and *** = highest variability in positive:negative FLAA test results. Pink zone shows the means below negative samples.

Following the same logic, frequencies of positive and negative results were plotted for each found substitution (Figure 9). Overall, the false-positive rate was 35% for actual negative samples detected by the FLAA test (none, red bar). DGPH was the only mutation resembling the expected RTqPCR proportion (all bars should have 100% and appeared in blue since all are positive for infection). Samples containing D2 and the Omicron variant had moderate false-negative rates (63:37 and 71:29, respectively).

In contrast, the ratio was higher in samples with DG alone or combined with TK (DG = 28:72 and DGTK = 20:80). Samples detected with three or four substitutions involving TA were the ones with the greatest variation in FLAA results, rendering 100% of false negatives (Figure 9, upper panel). Additionally, it was found that a high proportion of positive samples do have not any of these mutations (unidentified, ui). The dot-plots for the comparison of mean fluorescence showed that the lowest acquired readings were registered for TA-positive samples as well as DG-alone mutants as compared to the rest of the combinations and also that negative samples were placed below the overall average (Figure 9, lower panel). Mean nCts for all mutants were found in a range of 21 to 22 cycles and all are located above the overall average.

**Figure 10:**
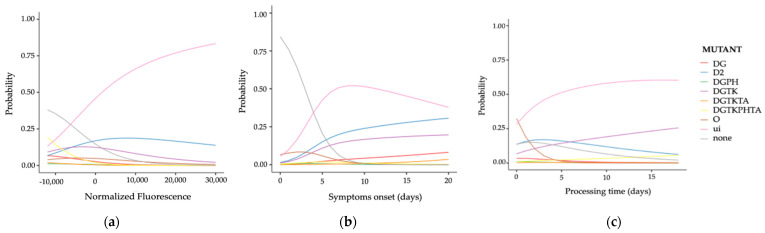
Estimated marginal means plots for (**a**) normalized fluorescence; (**b**) time for Symptoms onset and (**c**) time for sample processing.

On the other hand, the estimated marginal means plots were obtained by logistic regression for each mutation (Figure 10), which were contrasted by normalized fluorescence, and by times from symptoms onset and experimental processing. The gray curves on all graphs were taken as the reference probability for RTqPCR negative results, and it was assumed that any curve depicted below it meant a lower probability of detection. Noticeably, curves for TA were found under the reference curve at all plots (Figure 10a–c). It is worth mentioning that such mutations (TA) were found in samples measured through the indirect protocol, which in itself, presents high false-negative rates. Notwithstanding, when compared to Omicron samples that contain all mutations but TA, it can be seen that there is also a moderate alteration in the aptamer-mediated detection (ratio 71:29, Figure 10a), despite the fact that it was detected in samples from the direct reading experiments. This may mean that, regardless of readout type, mutations could had influenced FLAA results.

The combination of these results suggests a possible role for D614G and T732A mutations in the recognition of C7/C9 aptamers. DG mutation has been detected in the Omicron variant, and here, it showed a higher probability of detection compared with DG-alone samples. This suggest that samples with DG-alone mutation have other non-identified mutations affecting C7/C9 aptamers binding affinity and the combination of DG with other mutations only diminished it. On the contrary, TA substitution had a more detrimental effect since all of the samples result in 100% of false negatives, suggesting that this could be an essential aptatope for C7/C9 aptamers, but further investigation is required.

## 4. Discussion

Aptamer recognition has been a widely used biosensor development. Despite the recent advances in aptamer-based detection methods, most of the biosensors have been used to detect the S and N proteins or viral particles and have not been validated with clinical samples [25,26,27,28,29,30,31,32,33]. The calculated limit of detection (LOD) from several biosensors includes a wide range of protein S or N concentrations ranging from fM to nM, as previously described for C7/C9 biosensors. A few reported aptasensors with direct testing with clinical samples exhibit a wide range of sensitivity and specificity [15,16,17,18]. Some examples include high sensitivity and specificity electrochemical aptasensors. Singh et al. [15] presented an electrochemical aptasensor with a sensitivity and specificity of 100%. Zhang et al. [16] developed an electrochemical sensor with a sensitivity of 80.5% and specificity of 100% able to detect Alpha and Beta SARS-CoV-2 variants. In addition, Deng et al.’s [17] aptamer-based thermophoretic assay showed 100% sensitivity and specificity. Nevertheless, despite a high sensitivity and specificity, time-consuming sample preparation steps and/or expensive instrumentation are required for analysis.

For optical biosensors, the aptamer sandwich assay, developed by Svobodova et al. [18], resulted in 80% sensitivity and 40% specificity probably due to a background signal from the transport medium components. As described before, highly sensitive and specific biosensors based on aptamers have a promising potential as diagnostic agents.

However, all still require broader prospective studies that include a larger number of samples, the testing of optimal binding conditions, sample processing and acquisition timing and the SARS-CoV-2 variant testing. Thus, the aim of this work was to identify the best sampling conditions under a large clinical context and test in real-life conditions our C7/C9 aptasensor performance. As the number of aptasensors increases, we expect a rise of larger prospective studies for their diagnostics capabilities which can help to fulfill their potential commercial use in the long term.

Regarding the SARS-CoV-2 variants, Omicron has caused a huge concern owing to its higher contagion rate and vaccine-escape mutations [34] including those involved in aptamer recognition sites. The step-by-step analysis included here demonstrates that the C7/C9 aptasensor can be useful for SARS-CoV-2 Omicron detection. This C7/C9 aptasensor combination can be used alone or integrated with other sets of aptamers to develop a more selective signal depending on the SARS-CoV-2 variant detection. As this method could be used for large-scale sample processing, this is the first step to determine the assay performance for the screening, and diagnosis of different clinical samples across a range of clinical contexts and sample collection conditions.

## 5. Conclusions

In conclusion, we have depicted the performance of a fluorescence-linked aptamer assay as a diagnostic test for SARS-CoV-2. Real-life clinical conditions and a step-by-step statistical approach allowed us to find the best sample type, buffer, collection time and preparation protocol as well as the best cut-off point for ROC analysis. The determined conditions include sample collection from patients with symptomatology on set 4 days before sampling and an estimated processing time within 2 days of collection. The sample type included nasopharyngeal swabs preserved in aptamer buffer (TNa7) and the data acquisition had the best results when using the direct reading protocol with a cut-off point of −400 normalized fluorescence units for further ROC analysis. Moreover, RTqPCR mutations comparison with our FLAA test allowed us to preliminary propose possible aptatopes for C7/C9 aptamers, surpassing the need for the construction of multiple artificial mutations. Lastly, as this analysis allowed us to establish our best aptasensor conditions, this may serve as the base for a complete statistical methodology to establish optimal measurement conditions in diverse clinical samples for new diagnostic tests.

## Figures and Tables

**Table 1:** General characteristics of patients and collected samples.

	Frequency, N (%)		
	Total	Female	Male
Gender	242 (100)	131 (54)	111 (46)
Sal	158 (65)	90 (37)	68 (28)
Sw	35 (14)	19 (8)	16 (7)
Sw-D	49 (20)	22 (9)	27 (11)
Direct	164 (68)	84 (35)	80 (33)
Indirect	78 (32)	47 (19)	31 (13)
	**N**	**Mean**	**SD**
Age (years)	232	40.55	17.21
Symptoms onset (days)	213	5.65	4.64
Sample processing (days)	235	3.03	3.00

Sal = Saliva, Sw = Swab, Sw-D = Swab preserved in DMEM.

**Table 2:** Tetrachoric table for the determination of diagnostic parameters.

	RTqPCR		
FLAA	Positive	Negative	Total
Positive	92	25	117
Negative	82	43	125
Total	174	68	242
	**Parameters**		
Sensitivity		52.9%	
Specificity		63.2%	
Accuracy		55.8%	
Prevalence		71.9%	
Positive Predictive Value		78.6%	
Negative Predictive Value		34.4%	
Positive likelihood ratio		1.438	
Negative likelihood ratio		0.745	

**Table 3:** Summary of diagnostic accuracy parameters obtained by experimental settings.

	Sensitivity (%)	Specificity (%)	PPV (%)	NPV (%)	Youden Index	AUC	Cutoff Point
Sal	70.1	53.7	81.2	38.6	0.237	0.619	−103
Sw	**100**	**42.9**	**72.4**	**100.0**	**0.429**	**0.646**	**−398**
Sw-D	50	77.4	77.4	38.9	0.186	0.510	160
Indirect (10%)	54.7	72	80.6	42.9	0.267	0.655	−884
Direct (20%)	**93.3**	**25.0**	**77.2**	**57.9**	**0.183**	**0.531**	**−404**
Overall	71.1	50.7	78.3	41.2	0.218	0.587	−78

Bold numbers represent best cutoff points for FLAA testing.

**Table 4:** Summary of diagnostic accuracy parameters obtained by Ct range.

Ranges	Ct range	Sensitivity (%)	Specificity (%)	Accuracy(%)	AUC (%)
Overlapped	<20; 0–20	89	52	76	85
	<25	79	83	81	89
	<30	73	90	84	90
Independent	**20–25**	**90**	**80**	**86**	**91**
	**25–30**	**97**	**78**	**90**	**94**

Bold numbers represent best cutoff points for FLAA testing.

## Data Availability

Not applicable.

## Supplementary Materials

The following supporting information can be downloaded at: https://www.mdpi.com/article/10.3390/diagnostics12112829/s1, Figure S1: Agreement analysis for FLAA readings and RTqPCR Cts.; Figure S2: Dot-plots for the comparison of mean fluorescence and mean nCtOrf1ab.
