# Supplementary material for: Fluorescence-Linked Aptamer Assay for SARS-CoV-2 Spike-Protein: A Step-by-Step Performance Analysis in Clinical Samples
Source: Diagnostics (Basel). 2022 Nov 17;12(11):2829. doi: 10.3390/diagnostics12112829 (PMC9689637; doi:10.3390/diagnostics12112829)
Supplement: Supplementary file 1 [file diagnostics-12-02829-s001.zip › diagnostics-1954866-supplementary.pdf]

diagnostics

MDPI

SUPPLEMENTARY MATERIAL

In order to verify the internal agreement of readings, Bland-Altman analysis was performed. Results are shown in Supplementary Figure S1. Depiction of the results can be found in the subsection 3.1 of the result section within the manuscript.

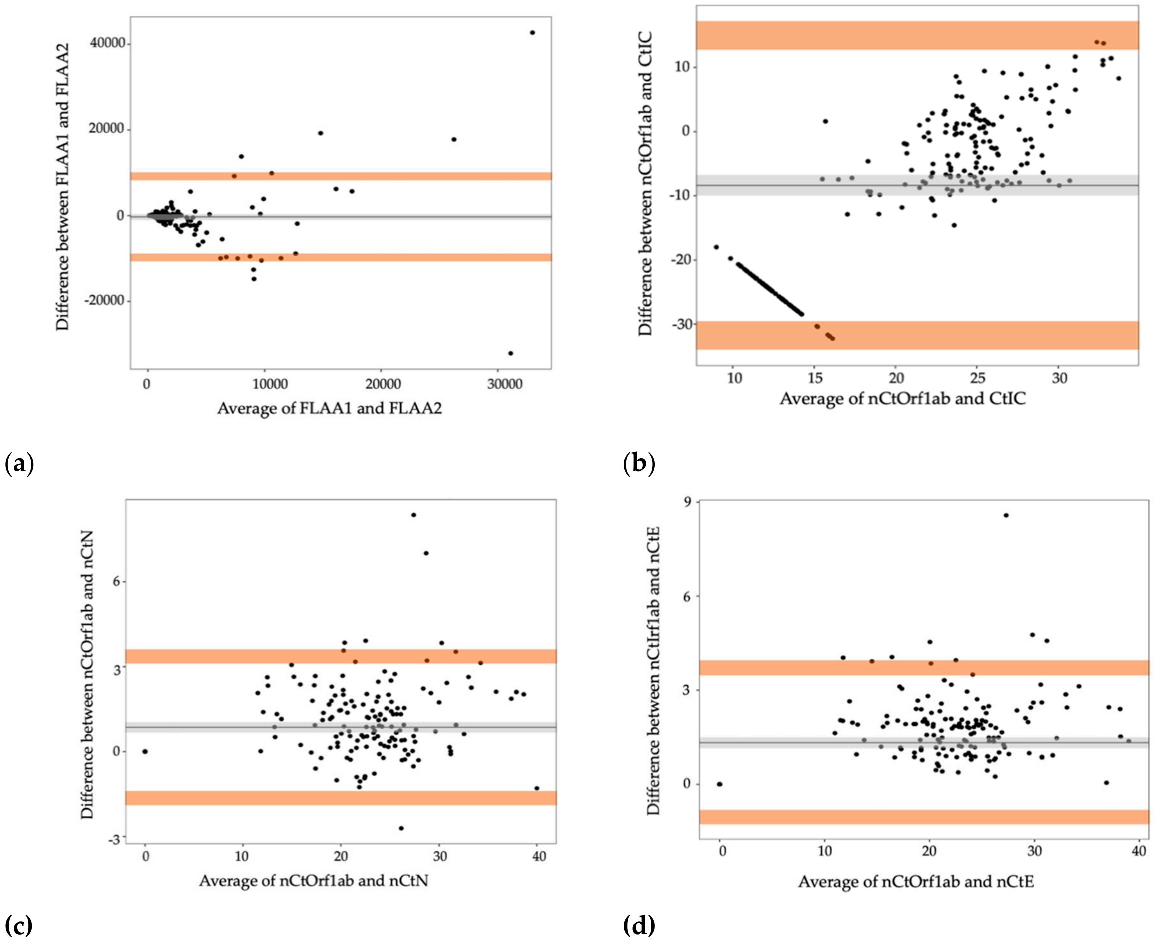

Supplementary Figure S1. Agreement analysis for FLAA readings and RTqPCR Cts. (**a**) Bland-Altman plot for FLAA1 and FLAA2 duplicates; (**b**) Bland-Altman plot for nCtOrf1ab vs CtIC. (**c**) Bland-Altman plot for nCtOrf1ab vs nCt of N gene; (**d**) Bland-Altman plot for nCtOrf1ab vs nCt of E gene.

To quantitatively compare data distribution, the means of normalized fluorescence from each experimental condition were visualized in dot-plots. Significant differences were found both in protocols and among sample types (Supplementary Figure S2).

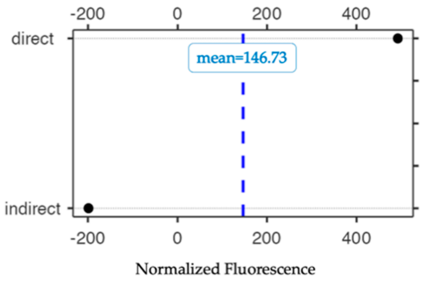

(**a**)

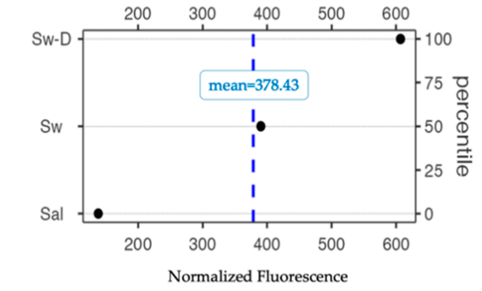

(**b**)

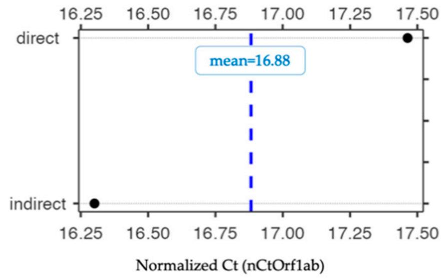

(c)

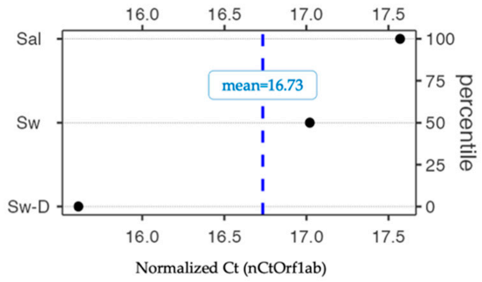

(d)

Supplementary Figure S2. Dot-plots for the comparison of (**a**) mean fluorescence by protocol; (**b**) mean fluorescence by sample type; (**c**) mean nCtOrf1ab by protocol and (**d**) mean nCtOrf1ab by sample type.
